# Mild Systemic Inflammation Increases Erythrocyte Fragility

**DOI:** 10.3390/ijms25137027

**Published:** 2024-06-27

**Authors:** Charlotte M. Stuart, Carmen Jacob, Aravinthan Varatharaj, Sarah Howard, Joe K. Chouhan, Jessica L. Teeling, Ian Galea

**Affiliations:** 1 Clinical Neurosciences, Clinical and Experimental Sciences, Faculty of Medicine, University of Southampton, Southampton SO16 6YD, UK; 2 Wessex Neurological Centre, University Hospital Southampton NHS Foundation Trust, Southampton SO16 6YD, UK; 3 Biological Sciences, Faculty of Life Sciences, University of Southampton, Southampton SO16 6YD, UK

**Keywords:** haemolysis, red blood cell, erythrocyte, fragility, inflammation

## Abstract

There is growing evidence that inflammation impairs erythrocyte structure and function. We assessed the impact of mild systemic inflammation on erythrocyte fragility in three different settings. In order to investigate causation, erythrocyte osmotic fragility was measured in mice challenged with a live attenuated bacterial strain to induce low-grade systemic inflammation; a significant increase in erythrocyte osmotic fragility was observed. To gather evidence that systemic inflammation is associated with erythrocyte fragility in humans, two observational studies were conducted. First, using a retrospective study design, the relationship between reticulocyte-based surrogate markers of haemolysis and high-sensitivity C-reactive protein was investigated in 9292 healthy participants of the UK Biobank project. Secondly, we prospectively assessed the relationship between systemic inflammation (measured by the urinary neopterin/creatinine ratio) and erythrocyte osmotic fragility in a mixed population (n = 54) of healthy volunteers and individuals with long-term medical conditions. Both human studies were in keeping with a relationship between inflammation and erythrocyte fragility. Taken together, we conclude that mild systemic inflammation increases erythrocyte fragility and may contribute to haemolysis. Further research is needed to assess the molecular underpinnings of this pathway and the clinical implications in inflammatory conditions.

## 1. Introduction

There is growing evidence that erythrocytes may be affected by inflammatory processes. Patients with COVID-19 have been found to have smaller and less deformable erythrocytes compared to controls, with differences still detectable four to eight months following hospital discharge [1]. Changes to erythrocyte deformability have also been observed in patients with other conditions associated with inflammation, such as obesity [2], cardiovascular disease [3], sepsis [4], and vitamin D status [5,6].

Particularly in sepsis models, a number of pathways have been described through which inflammation may impact erythrocytes, such as mechanical factors, energy deficiency, and oxidative stress. Amongst mechanical factors, vascular changes during systemic inflammation can lead to microangiopathic haemolysis [7,8]. Reduced capillary flow and microvascular stasis, as seen in excessive inflammation associated with sepsis, increase erythrocyte fragility through mechanical and enzymatic cell damage [8,9]. Higher glucose consumption by immune processes during inflammation can severely restrict glucose availability [10,11]. Energy deficiency causes an increase in intracellular calcium ions, resulting in decreased deformability [12] and likely further changes, such as modulation of signals associated with erythrocyte clearance (leaflet phosphatidylserine distribution), morphology, and adhesion [4]. Most importantly, inflammation leads to oxidative stress, which, in turn, increases erythrocyte membrane rigidity and therefore reduces deformability [13]. Erythrocytes have an elaborate multi-dimensional defence system to protect themselves against oxidative stress [14], mainly because they contain a huge payload of haemoglobin which in itself is a potent oxidising agent. This defence system consists of a range of antioxidant enzymes (e.g., superoxide dismutase, glutathione peroxidase, glutathione reductase, peroxiredoxin 2, thioredoxin and its reductase, and catalase) as well as small molecules (e.g., glutathione, nicotinamide adenine dinucleotide phosphate, ascorbate, and tocopherol). As erythrocytes travel through the immediate tissue microenvironment of inflammatory foci characterised by neutrophil and/or macrophage oxidative burst activity, it is conceivable that the erythrocyte’s auto-protective systems are unable to deal with the sudden oxidative load, leading to lipid and protein oxidation, damage to internal structures and membrane, a decrease in membrane fluidity and finally haemolysis. Once the erythrocyte’s antioxidative defences are overwhelmed, haemoglobin may amplify this process since it is capable of auto-oxidation, a process that generates superoxide radicals that can no longer be neutralised.

Erythrocyte haemolysis is of particular interest as erythrocytes contain vast amounts of haemoglobin, which is highly toxic in its extracellular, unbound form [15]. Upon release from the erythrocyte, haemoglobin is immediately bound by haptoglobin, a very abundant plasma protein with an extremely high affinity for haemoglobin. This binding neutralizes haemoglobin toxicity, and the complex is cleared by CD163-mediated endocytosis. Damaged haemoglobin may release its toxic moiety haem, and a second-line system exists to scavenge haem by binding to haemopexin, followed by CD91-mediated clearance. If these systems are overwhelmed, free haemoglobin and haem have well-documented, dose-dependent systemic effects, most notably nitric oxide depletion, which causes platelet activation and smooth muscle contraction, leading to a variety of symptoms, including abdominal pain, dysphagia, arterial and pulmonary hypertension, and erectile dysfunction [16]. Kidney injury follows intravascular haemolysis via several pathways, such as oxidative stress, cytotoxicity, and direct and indirect pro-inflammatory signals [17]. Haem is a potent inflammatory stimulus, activating the innate immune system via multiple pathways. Administration of haem to mice resulted in increased vascular permeability, adhesion molecule expression, and leukocyte migration [18]. Also, haem acts as a danger-signalling damage-associated molecular pattern which binds to toll-like receptor 4 [19], stimulates neutrophil activation [20], leads to neutrophil extracellular trap formation [21], and activates the alternative complement pathway [22]. Haemolysis is thought to contribute to the pathogenesis of sepsis [8,23] and severe COVID-19 [24], and hence has been proposed as a new treatment target [24]. Due to its pro-inflammatory properties, extracellular haemoglobin may also be associated with atherosclerotic plaque instability [25]. Associations between extracellular haemoglobin levels and disease progression have also been demonstrated in neurological conditions such as multiple sclerosis [26].

Milder degrees of systemic inflammation may not cause haemolysis but be sufficient to induce external erythrocyte membrane damage, leading to reduced deformability. Changes to deformability and and erythrocyte morphology, such as increased red cell distribution width, have been reported in association with chronic conditions associated with systemic inflammation [2,3,27,28], but whether this is causative remains to be shown. While the effects of sepsis on erythrocytes have been studied [4], the consequences of low-grade systemic inflammation on erythrocyte fragility and haemolysis are not known.

Here, we investigate the hypothesis that mild systemic inflammation contributes to erythrocyte fragility. In a murine model, we tested the effects of a mild inflammatory challenge (with attenuated *Salmonella typhimurium*) and found that it increased erythrocyte fragility to an osmotic challenge (Study 1). Then, using retrospectively collected UK biobank data, we analysed the relationship between haemolytic and inflammatory parameters in a healthy human population and found a significant association (Study 2). Finally, we assessed the relationship between systemic inflammation and erythrocyte osmotic fragility in healthy volunteers and patients with a range of non-haematological long-term medical conditions in a prospective observational study, and confirmed an association (Study 3).

## 2. Results

### 2.1. Study 1—Murine Model

**Figure 1:**
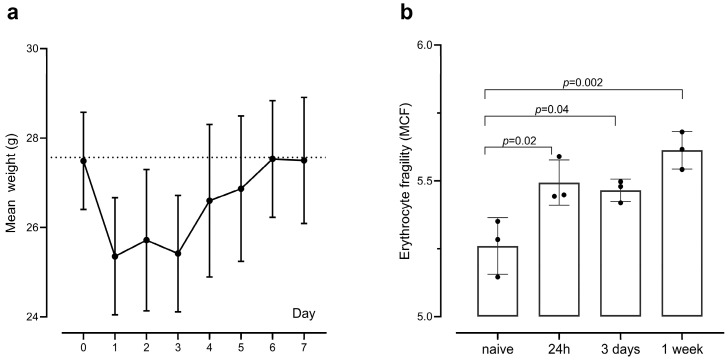
A mild systemic inflammatory stimulus increases erythrocyte fragility in mice. C57BL/6 mice received an intraperitoneal challenge with attenuated *Salmonella typhimurium* vaccine strain SL3261, and their erythrocyte fragility, measured in an ex vivo osmotic fragility assay, was compared at three time points (n = 3 each time point) versus naive mice (n = 3). (**a**) Daily weight of mice receiving the bacterial challenge. (**b**) Erythrocyte fragility as quantified by the median corpuscular fragility (MCF) (n = 3 each time point). Data are shown as mean ± SD.

This experimental study in mice aimed to test the hypothesis that systemic inflammation leads to erythrocyte fragility. Intraperitoneal challenge with an attenuated form of *Salmonella typhimurium* was used as a model of mild systemic inflammation, which is prolonged yet short-lived [29,30]. The cytokine profile of this experimental model has been described elsewhere [30]. This model was selected over single challenges with lipopolysaccharides or cytokines since it more closely recapitulates naturally occurring mild systemic inflammation. Erythrocyte fragility was measured using an osmotic lysis assay and quantified as the median corpuscular fragility (MCF), which is the concentration of NaCl causing 50% haemolysis. Mice challenged with *Salmonella* did not become unwell but nevertheless developed weight loss at 24 h (Figure 1a; mean weight loss −7.78%, *t* (8) = 12.20; *p* < 0.001, r = 0.973), which they recovered within the next few days such that on day 6, their weight was not significantly different compared to baseline. Such a mild clinical picture is characteristic of the challenge with this attenuated strain of *Salmonella typhimurium* [30,31]. A one-way ANOVA was conducted to compare the erythrocyte fragility (as quantified by the MCF) of the *Salmonella*-challenged animals at each time point with that of the control mice (n = 3 per group). There was a significant difference in MCF between the bacterial challenge and control groups (F (3,8) = 10.57, *p* = 0.0037). Bonferroni post hoc test showed that erythrocytes from challenged mice demonstrated increased fragility at each time point compared with the control mice (24 h: mean = 5.494, *p* = 0.0191; 3 days: mean = 5.4650, *p* = 0.0370; 1 week: mean = 5.6127, *p* = 0.0017; Figure 1b).

### 2.2. Study 2—A Retrospective Observational Clinical Study

#### 2.2.1. Reticulocytes Are Valid Markers of Haemolysis

Having determined that systemic inflammation leads to erythrocyte fragility, we next wanted to seek evidence for this phenomenon in humans. Short of a human experimental study, we sought observational evidence of an association between systemic inflammation and erythrocyte fragility and identified the UK Biobank as a large cohort of individuals in whom systemic inflammation was measured with a high-sensitivity C-reactive protein (hsCRP) assay with reticulocyte studies performed on the same day. The number of reticulocytes, the precursors of erythrocytes, increases in response to erythrocyte loss, which occurs during haemolysis. Three reticulocyte parameters were assessed as primary surrogate outcome measures for haemolysis: reticulocyte percentage (Ret%); high-light-scatter reticulocyte percentage (iRet%), which corresponds to the most immature reticulocytes; and the immature reticulocyte fraction (IRF) calculated by dividing the number of immature reticulocytes by the total reticulocytes. First, the three reticulocyte measures (Ret%, iRet%, and IRF) were validated as surrogate markers of haemolysis in UK Biobank participants with haemolytic anaemias (n = 1740) versus control healthy individuals (n = 9738). A main-effects ANCOVA, correcting for age and sex, showed that all three reticulocyte measures were significantly higher in people with haemolytic anaemias (Table 1).

#### 2.2.2. Reticulocytes and High-Sensitivity C-Reactive Protein

Out of the 9738 individuals with available reticulocyte data, 9292 had a hsCRP assay performed at the same time. Multivariable linear regression was carried out to determine the relationship between the three reticulocyte parameters and hsCRP in healthy participants (n = 9292). Adjustment for age and sex was performed since both of these variables may affect inflammation [32] and reticulocytes [33], and we wanted to study the effect of systemic inflammation that was independent of age and sex. The results of these analyses are summarised in Table 2. A positive association was observed between hsCRP and each reticulocyte parameter. The standardised beta coefficients show that hsCRP was more strongly predictive of the three surrogate markers of haemolysis than either age or sex.

The reference range for the hsCRP assay had a lower limit of 1 mg/L, but its lower limit of quantification was 0.08 mg/L. Hence, while individuals with hsCRP < 1 mg/L are classified as having a normal CRP for clinical purposes, the assay’s dynamic range extended below this level, providing quantification of subclinical degrees of systemic inflammation occurring in healthy individuals. To investigate whether the association between reticulocyte markers of haemolysis and hsCRP was also seen at these very mild degrees of systemic inflammation, a sensitivity analysis was conducted in those with hsCRP < 1 mg/L (n = 5037). This yielded similar results (Table 3).

### 2.3. Study 3—A Prospective Observational Clinical Study

#### 2.3.1. Rationale and Study Population

Although the UK Biobank study demonstrated that systemic inflammation was associated with surrogate reticulocyte markers of haemolysis, it had severe limitations: the design was retrospective, erythrocyte fragility was not directly assessed, and a single hsCRP estimation in serum does not fully represent an individual’s fluctuations in systemic inflammatory status over time. Hence, the next step was to prospectively assess erythrocyte fragility alongside an integrative marker of exposure to systemic inflammation. Resilience to osmotic stress was selected as a readout of erythrocyte fragility given its widespread and accepted use in human clinical studies [34] and to act as a parallel to the murine experimental Study 1 above. The urinary neopterin-to-creatinine ratio (UNCR) was employed to integrate the individual’s exposure to systemic inflammation over the previous day [32]. Neopterin is secreted by myeloid lineage cells during any type of inflammation and rapidly enters the circulation to be excreted in the urine. Normalization of urinary neopterin to urinary creatinine removes variations due to hydration status and creatinine clearance. An early morning urine sample integrates systemic inflammation over the preceding twenty-four hours. Participants (n = 54) had a mean age of 43.3 years (SD: 13.5, range: 19–81 years), 69.8% were female, 68.2% had no comorbidities, and 71.4% were not on any regular medication.

#### 2.3.2. Systemic Inflammation Correlates with Erythrocyte Fragility

A multivariable linear regression was performed to assess the relationship between UNCR (systemic inflammation) and MCF (erythrocyte fragility), adjusting for age and sex. The model significantly predicted MCF (F(3,50) = 10.081, *p* < 0.0001, R^2^ = 0.339; Table 4) and accounted for 33.9% of the variance observed. UNCR was significantly associated with MCF. Sensitivity analyses excluding participants with comorbidities resulted in loss of statistical power but showed a similar trend (Table 4).

## 3. Discussion

The studies presented here support the hypothesis that mild systemic inflammatory stimuli lead to increased erythrocyte fragility. This was demonstrated in mice (Study 1) and supported by observations made in humans (Studies 2 and 3). Two remarkable observations in the human studies deserve mention. In Study 2, systemic inflammation had a larger effect size on reticulocyte parameters than age and sex, which are well-established determinants of reticulocyte turnover [33]. In Study 3, the effect size of UNCR was similar to that of age, and the combination of age and UNCR was able to together explain 33.9% of the variance in the MCF. Overall, the studies are in keeping with mild systemic inflammation leading to erythrocyte fragility. While it is well-documented that severe inflammation during sepsis affects erythrocyte morphology and function [4], the studies presented here provide new insights into the effects of mild and even subclinical levels of inflammation on erythrocyte health. We also establish a causative role for systemic inflammation in decreasing erythrocyte resilience and increasing susceptibility to haemolysis. This finding is an important contribution to the literature since it implies the possibility of positive feedback whereby inflammation can cause haemolysis and, in turn, the main products of haemolysis, namely haemoglobin and haem, can increase inflammation. As discussed in the introduction, systemic inflammation may cause erythrocyte fragility through a variety of mechanisms, but further study is required to map the underlying cellular and molecular mechanisms.

Since direct experimental interrogation to prove causation is difficult in humans, Studies 2 and 3 were aimed at gathering observational evidence that the effect of mild systemic inflammation on erythrocyte fragility is also relevant to humans. In Study 2, hsCRP was used as an indicator of systemic inflammation in healthy participants. A total of 98.4% of participants in this study had hsCRP levels not suggestive of an acute phase response (i.e., ≤10 mg/L; [35]). Mild to moderate increases in hsCRP (1 mg/L to 10 mg/L) have been found to be associated with increased cardiovascular risk [35] and are thought to correlate with so-called metabolic inflammation [36]. Other conditions thought to be associated with moderate elevation of C-reactive protein levels are viral infections, low-grade mucosal infections (e.g., periodontitis), obesity, insulin resistance, depression, smoking, and mild alcohol consumption [36]. Interestingly, the predictive effect of hsCRP on surrogate haemolytic markers was retained when restricting the analysis to participants with clinically “normal” levels of hsCRP (<1 mg/L). While Study 2 relied on surrogate markers of haemolysis, Study 3 was planned to directly assess erythrocyte fragility while at the same time measuring the individual’s exposure to systemic inflammation using UNCR [32].

This work’s major strength is that the results of all three studies support the hypothesis that erythrocyte fragility increases in the presence of mild systemic inflammation. This consistency across three different contexts, including a murine model and human observational study, and with different methodologies, demonstrates internal validity and provides an acceptable degree of confidence. We also highlight the potential utility of reticulocyte measures—these parameters are easily acquired on most commercially available automated haematology analysers used worldwide. While manual osmotic fragility testing is laborious, semi-automated instruments are now available to measure this marker using osmotic gradient ektacytometry.

There are some limitations which are highlighted here. While UNCR integrates inflammation over the preceding 24 h and is an improvement on hsCRP, which only gives information about the inflammatory status at a certain point in time, UNCR also varies from week to week [32]. However, studies on erythrocyte deformability following COVID-19 suggest that changes in erythrocyte deformability (and therefore possibly also fragility) persist over months, so the effects of inflammation on erythrocytes appear to be stable [1].

By necessity, Study 2 was restricted to employing surrogate markers of haemolysis since direct measures of haemolysis were not acquired in this retrospective study. Whilst our analyses on study participants with haemolytic anaemias supported the use of reticulocyte parameters as surrogate markers of haemolysis, it is likely that other factors apart from haemolysis influence reticulocyte parameters. For example, direct effects of inflammation on erythropoiesis as a form of stress erythropoiesis have been proposed [37]. Hence, it is possible that inflammation in Study 2 was linked to reticulocyte parameters via stress erythropoiesis rather than erythrocyte fragility. This is somewhat mitigated by the direct assessment of erythrocyte fragility in Studies 1 and 3.

Osmotic fragility testing is a widely used method to analyse erythrocyte fragility [34]. Yet osmotic stress is not encountered physiologically. While the physical and biochemical factors that cause osmotic and mechanical fragility are thought to be similar [38], future studies could utilise other more physiologically relevant methods to assess red blood cell resilience, such as ektacytometry.

Recent or regular exercise can impact erythrocyte fragility and erythropoiesis and was not controlled for in Studies 2 and 3. “Acute” exercise involves several stressors that lead to decreased deformability and mechanical rupture of erythrocytes with subsequent increases in erythropoiesis straight afterwards, whilst regular exercise leads to an overall improvement in erythrocyte integrity [39]. As we investigated a large sample of participants in Study 2, it is unlikely that different levels of exercise in individuals led to significant bias.

The present studies do not allow one to draw conclusions on how long changes in erythrocyte fragility persist following an inflammatory stimulus and whether the increase in erythrocyte fragility as assessed by MCF actually contributes to in vivo haemolysis. However, given the relatively long lifespan of erythrocytes, it is likely that changes to erythrocyte fragility are cumulative. Longitudinal studies are needed to clarify the evolution of erythrocyte fragility alongside exposure to systemic inflammation.

When considering the potential clinical impact of low-grade intravascular haemolysis, the role of haemoglobin and haem scavenging mechanisms should not be overlooked. Haptoglobin, for example, is known to be elevated during infection as an acute phase reactant [40] and might be able to counteract the potential effects of low-grade intravascular haemolysis. Interestingly, certain haptoglobin phenotypes have been associated with an increased risk of inflammation and cardiovascular and auto-immune disease [41,42]. Future studies should measure serum haptoglobin and haemopexin, as well as haptoglobin genotype.

In the absence of inflammation, severe haemolysis overwhelming the capacity of haptoglobin/haemopexin scavenging systems may result in high serum iron and iron deposition in multiple organs, especially in the kidney [43]. Although we have not studied iron metabolism in the present work, it would be interesting to explore this in future studies. The situation would be expected to be complex during haemolysis in the context of systemic inflammation when iron metabolism is dysregulated. Typically, immune activation causes an “iron withdrawal“ whereby iron absorption is reduced and shuttled away from haematopoiesis to the innate immune compartment, resulting in anaemia of inflammation [44]; besides providing macrophages and neutrophils with iron needed for respiratory chain and oxidative burst enzymes, reduced availability of iron (i.e., low serum iron) is protective during infection [44]. Hence, iron deposition in target organs during mild systemic inflammation appears unlikely, but this hypothesis requires testing. The situation would be expected to be different in the presence of focal inflammation in local tissues, which may result in local deposition of iron because of macrophage/neutrophil infiltration, microvascular damage leading to extravasation of blood, or disturbed tissue-specific iron handling. Hence, it is not uncommon to observe local tissue iron deposition in chronic disease conditions such as atherosclerosis [45], various types of arthritis [46], multiple sclerosis [47], and chronic liver diseases [48].

The clinical significance of increased erythrocyte fragility and haemolysis in people with long-term inflammatory conditions needs to be studied further. A study in people with progressive multiple sclerosis suggests an association between neurodegeneration and levels of free haemoglobin, as brain atrophy rate in patients correlated with free haemoglobin levels [26]. Our study provides a possible underlying mechanism since systemic and/or organ-specific inflammation may increase erythrocyte fragility, leading to higher levels of free haemoglobin in individuals with more rapidly progressive multiple sclerosis. It seems likely that inflammation-driven low-grade haemolysis might drive progression in neurological and other long-term conditions, but this requires further investigation.

## 4. Methods

### 4.1. Study 1—Murine Model

#### 4.1.1. Setup

Adult male C57BL/6 mice (n = 12, 10 weeks old) were housed in groups of three in plastic cages with sawdust bedding. Food and water (standard chow, RM1, SDS, UK) were available *ad libitum*. The holding room was temperature controlled (19 to 23 °C) with a 12 h light followed by a 12 h dark cycle (light on at 07:00 h). Mice were allowed to acclimatise to the holding room for 1 week before the start of the experiment. All procedures were performed under UK Home Office project licence P4155EEE0 and in accordance with the United Kingdom Animals (Scientific Procedures) Act (1986).

#### 4.1.2. Challenge with Salmonella typhimurium

At 10 weeks of age, mice (n = 9) were administered a single intraperitoneal injection (200 µL) of 106 colony-forming units (cfu) of attenuated *Salmonella typhimurium* vaccine strain SL3261 (generously provided by Dr. H. Atkins, DSTL, Salisbury, UK) to induce systemic inflammation. With this vaccine strain of *Salmonella*, replicative infection does not occur, and mice look outwardly well; it models a period of prolonged low-grade systemic inflammation lasting several days [29,30]. Three mice acted as negative controls. Body weight was recorded at baseline and daily after injection. Mice challenged with *Salmonella* were sacrificed at 1, 3, and 7 days after challenge (n = 3 on each day).

#### 4.1.3. Murine Sacrifice and Blood Collection

Mice were terminally anaesthetised with an intraperitoneal injection of Avertin (2,2,2-tribromoethanol 3% *w*/*v* and 1.8% 2-methyl-2-butanol (Sigma Aldrich) in 7.2% alcohol diluted with 0.9% saline). After sacrifice, the thorax of the mouse was exposed, and 3 drops of heparin sodium (5000 international units per mL) were added to the cavity to prevent clotting. The left atrium was punctured, and blood was collected from the chest cavity using a 1 mL syringe and transferred to a heparin sodium-coated microcentrifuge tube. The blood was immediately used for erythrocyte osmotic fragility tests.

#### 4.1.4. Osmotic Fragility Testing

Osmotic stress was applied using Parpart’s method [49]. Briefly, 20 μL of whole blood was suspended for 30 min in 1 mL NaCl-PO_4_ (Parpart’s buffered saline, Clin-Tech, Guildford, UK) at increasing concentrations with total cell lysis occurring at 0 g/L. The cells were then centrifuged at 1700× *g* for 5 min at room temperature, and the haemoglobin concentration in the supernatant was determined by its optical density (OD) at 540 nm. The haemolysis level at each osmotic stress level was expressed as % of the total lysis, according to the following formula:

% haemolysis = 100 × [(ODS − OD_0_)/(ODT − OD_0_)]

where OD_0_ = OD of 9.0 g/L saline, ODS = OD of supernatant, and ODT = OD of supernatant from erythrocytes subjected to total haemolysis. A four-parameter variable slope sigmoidal model was fitted to the data in GraphPad Prism v8 and used to determine the IC50, which is the concentration of NaCl causing 50% haemolysis (also known as the median corpuscular fragility, MCF). The normal range of MCF at pH 7.4 and 20 °C is 4.0 to 4.45 g/L [49].

#### 4.1.5. Outcome Parameters

The primary outcome was MCF. Body weight was also assessed.

### 4.2. Study 2—A Retrospective Observational Clinical Study

#### 4.2.1. Participants

This project was approved by UK Biobank (approval number 61771). Between 2006 and 2010, approximately 500,000 people between the ages of 40 and 69 years were recruited to participate in the UK Biobank project (www.ukbiobank.ac.uk, accessed on 26 June 2024). Participants with available reticulocyte parameter data, who did not have any recorded diagnosis in the “First occurrence“ data category number 1712 (which encodes the first occurrence of health conditions defined by their 3-character ICD10 code in any healthcare setting) and any recorded inpatient diagnosis after hospitalisation, and who did not take any regular medication, were included as healthy participants (n = 9738).

#### 4.2.2. Outcome Parameters

We investigated the utility of reticulocyte parameters as surrogate markers of haemolysis. Reticulocytes are the immature precursors of erythrocytes, which increase in response to erythrocyte clearance. The percentage of reticulocytes to total erythrocytes in the peripheral blood indicates the rate of erythrocyte turnover, whilst the number (a raw count) of reticulocytes reflects the amount of erythropoiesis on a given day. UK Biobank participants had a full blood count, including counts of total and high-light-scatter reticulocytes.

For the purposes of the analysis here, three reticulocyte parameters were assessed as primary surrogate outcome measures for haemolysis: reticulocyte percentage (Ret%), high-light-scatter or immature reticulocyte percentage (iRet%), and immature reticulocyte fraction (IRF) calculated by dividing the number of immature reticulocytes by the total reticulocytes.

These reticulocyte parameters were first validated as surrogate markers of haemolysis in UK Biobank participants (n = 1740) with a diagnosis of haemolytic anaemia including thalassaemia (ICD-D56), sickle-cell disorders (ICD-D57), acquired haemolytic anaemia (ICD-D59), and other hereditary haemolytic anaemias (ICD-D58), versus control healthy individuals (n = 9738).

High-sensitivity C-reactive protein in serum was used as a marker of systemic inflammation.

#### 4.2.3. Sample Collection and Analysis

Blood samples analysed at the baseline assessment were used for the present analysis since this represented the largest number of samples. Blood samples were collected in 4 mL EDTA vacutainers and analysed within 24 h. Reticulocyte parameters were acquired on an automated Beckman Coulter LH 750 Haematology Analyser. For hsCRP analysis, blood was collected in a silica clot accelerator (SST) vacutainer and allowed to clot at room temperature for 30 min. Serum aliquots were stored at −80 °C until analysed for hsCRP by immunoturbidimetry. The dynamic range of the assay was 0.08–80 mg/L. Samples above the upper limit of quantification were re-run after dilution. Reticulocyte parameters and hsCRP were available in a common dataset of 9292 healthy participants.

### 4.3. Study 3—A Prospective Observational Clinical Study

#### 4.3.1. Participants

Adult participants were recruited and consented (national research ethics approvals 12/SC/0176 and 18/LO/2105; institutional research ethics approvals ERGO5562 and ERGO46018). Exclusion criteria for control individuals were (1) haematological disorders, (2) autoimmune or active inflammatory disorders, and (3) chronic medical conditions that were uncontrolled or active at the time of recruitment. All participants were non-smokers in the year preceding study participation.

#### 4.3.2. Outcome Parameters

The primary outcome parameter was MCF.

#### 4.3.3. Sample Collection

For the erythrocyte osmotic fragility tests, whole blood was collected in 6 mL K2-EDTA vacutainer tubes (BD, UK). An additional 3 mL K2-EDTA sample was collected for a full blood count, performed on a Sysmex XN10 analyser in Southampton General Hospital‘s haematology laboratory. Great care was taken to reduce the chance of in vitro haemolysis of the samples by careful handling and transport of samples in a padded container. Erythrocyte osmotic fragility testing was performed within 15 min of venesection.

The UNCR was measured using liquid chromatography followed by mass spectrometry; this method has been validated as a marker of systemic inflammation [32]. Participants were asked to collect the first (midstream) urine sample passed in the morning and were instructed to protect the sample from light using aluminium foil since neopterin is photosensitive. Urine specimens were centrifuged at 10 °C and 2000× *g* for five minutes and stored at −80 °C in 1 mL aliquots. The detailed protocol for UNCR measurement has been previously described [32].

#### 4.3.4. Osmotic Fragility Testing

Osmotic fragility testing was performed as detailed in Section 4.1.4.

### 4.4. Statistical Analyses

SPSS v28 was used for statistical analyses, and plots were created in GraphPad Prism v9. Data distribution was determined graphically and using the Kolmogorov–Smirnov test. Parametric or non-parametric tests were used where appropriate. All hypothesis testing was two-tailed. A *p*-value < 0.05 was considered significant. Mean ± standard deviation (SD) is quoted unless otherwise stated.

## Figures and Tables

**Table 1:** Validation of reticulocyte parameters as surrogate markers of haemolysis. Analysis of covariance examining reticulocyte markers between healthy participants (n = 9738) and those with haemolytic anaemias (n = 1740), adjusting for age and sex (model F (3, 11,474) = 55.467, *p* < 0.001). Abbreviations: Ret%, reticulocyte percentage; iRet%, high-light-scatter or immature reticulocyte percentage; IRF, immature reticulocyte fraction.

		Ret%	iRet%	IRF
**Estimated Marginal Means**	**Healthy controls**	1.274	0.356	0.276
	**Haemolytic anaemia**	1.647	0.568	0.330
**Significance (*p*)**		<0.0001	<0.0001	<0.0001
**Partial eta squared**		0.13	0.065	0.084

**Table 2:** The association between systemic inflammation and surrogate markers of haemolysis in healthy individuals (retrospective study). The results are from three multivariable linear regression analyses showing the relationship between systemic inflammation (independent predictor variable: high-sensitivity C-reactive protein) and reticulocyte-based surrogate haemolytic markers (dependent variable in each regression: Ret%, iRet%, or IRF) while correcting for age and sex in healthy participants (n = 9292). Sex was coded as 1 for males and 0 for females. Abbreviations: Ret%, reticulocyte percentage; iRet%, high-light-scatter or immature reticulocyte percentage; IRF, immature reticulocyte fraction; hsCRP, high-sensitivity C-reactive protein; CI, confidence interval.

Dependent Variable			Ret%	iRet%	IRF
**Model**	**F statistic**		13.017	55.167	51.227
	**Degrees of freedom**		3, 9288	3, 9288	3, 9288
	**Significance (*p*)**		<0.0001	<0.0001	<0.0001
	**R^2^ (corrected)**		0.004	0.017	0.016
**Variables**	**Standardised beta coefficients**	**Age**	−0.009	−0.030	−0.014
		**Sex**	0.038	0.042	−0.009
		**hsCRP**	0.050	0.120	0.127
	**Unstandardised coefficients** **(95% CI)**	**Age**	−0.001 (−0.005, 0.002)	−0.001 (−0.001, 0)	−0.0001 (0, 0)
		**Sex**	0.89 (0.041, 0.137)	0.018 (0.009, 0.026)	−0.001 (−0.004, 0.001)
		**hsCRP**	0.021 (0.013, 0.030)	0.009 (0.008, 0.011)	0.003 (0.002, 0.003)
	**Significance (*p*)**	**Age**	0.379	0.005	0.193
		**Sex**	<0.0001	<0.0001	0.367
		**hsCRP**	<0.0001	<0.0001	<0.0001

**Table 3:** The association between systemic inflammation and surrogate markers of haemolysis in healthy individuals with low levels of high-sensitivity C-reactive protein (retrospective study). The results are from three multivariable linear regression analyses showing the relationship between systemic inflammation (independent predictor variable: high-sensitivity C-reactive protein) and surrogate haemolytic markers (dependent variable in each regression: Ret%, iRet%, or IRF) while correcting for age and sex in healthy participants with high-sensitivity C-reactive protein < 1 mg/L (n = 5037). Sex was coded as 1 for males and 0 for females. Abbreviations: Ret%, reticulocyte percentage; iRet%, high-light-scatter or immature reticulocyte percentage; IRF, immature reticulocyte fraction; hsCRP, high-sensitivity C-reactive protein; CI, confidence interval.

Dependent Variable			Ret%	iRet%	IRF
**Model**	**F statistic**		20.941	52.785	24.325
	**Degrees of freedom**		3, 5033	3, 5033	3, 5033
	**Significance (*p*)**		<0.0001	<0.0001	<0.0001
	**R^2^ (corrected)**		0.012	0.030	0.014
**Variables**	**Standardised beta coefficients**	**Age**	−0.052	−0.071	−0.030
		**Sex**	0.046	0.037	−0.010
		**hsCRP**	0.086	0.158	0.119
	**Unstandardised coefficients** **(95% CI)**	**Age**	−0.006 (−0.009, −0.003)	−0.002 (−0.002, −0.001)	−0.0002 (−0.0005, 0)
		**Sex**	0.074 (0.029, 0.118)	0.013 (0.003, 0.022)	−0.001 (−0.004, 0.002)
		**hsCRP**	0.290 (0.197, 0.348)	0.115 (0.095, 0.135)	0.029 (0.022, 0.036)
	**Significance (*p*)**	**Age**	<0.001	<0.0001	0.037
		**Sex**	0.0012	0.008	0.482
		**hsCRP**	<0.0001	<0.0001	<0.0001

**Table 4:** The association between systemic inflammation and median corpuscular fragility (prospective study). The results are from two multivariable linear regression analyses showing the relationship between systemic inflammation (independent predictor variable: urinary neopterin-to-creatinine ratio) and median corpuscular fragility while correcting for age and sex. The first regression was a complete case analysis of all participants (n = 54); the second regression was a sensitivity analysis excluding participants with comorbidities (n = 35). Sex was coded as 1 for males and 0 for females. Abbreviations: MCF, median corpuscular fragility; UNCR, urinary neopterin-to-creatinine ratio; CI, confidence interval.

			All Participants (n = 54)	No Comorbidities (n = 35)
**Model**	**F statistic**		10.081	4.454
	**Degrees of freedom**		3, 50	3, 31
	**Significance (*p*)**		<0.0001	0.0102
	**R^2^ (corrected)**		0.339	0.234
**Variables**	**Standardised beta coefficients**	**Age**	0.441	0.456
		**Sex**	−0.123	0.018
		**UNCR**	0.353	0.296
	**Unstandardised coefficients** **(95% CI)**	**Age**	0.008 (0.004, 0.012)	0.008 (0.003, 0.014)
		**Sex**	−0.063 (−0.181, 0.055)	0.009 (−0.142, 0.159)
		**UNCR**	0.001 (0.001, 0.002)	0.001 (0, 0.002)
	**Significance (*p*)**	**Age**	<0.001	0.005
		**Sex**	0.288	0.906
		**UNCR**	0.004	0.066

## Data Availability

Data are available on request subject to ethical and institutional approvals.
